# Aspiration thrombectomy of M2 middle cerebral artery occlusion to treat acute ischemic stroke: A core lab–adjudicated subset analysis from the COMPLETE registry and literature review

**DOI:** 10.3389/fneur.2023.1076754

**Published:** 2023-03-30

**Authors:** Johanna T. Fifi, Kurt Yaeger, Stavros Matsoukas, Ameer E. Hassan, Albert Yoo, Sunil Sheth, Osama O. Zaidat

**Affiliations:** ^1^Department of Neurosurgery, Icahn School of Medicine at Mount Sinai, New York, NY, United States; ^2^Valley Baptist Medical Center, Department of Neurology, University of Texas Rio Grande Valley, Harlingen, TX, United States; ^3^Texas Stroke Institute, Dallas, TX, United States; ^4^UTHealth McGovern Medical School, Houston, TX, United States; ^5^Department of Endovascular Neurosurgery, Mercy Health St. Vincent Medical Center, Toledo, OH, United States

**Keywords:** stroke, CT angiography, thrombectomy, middle cerebral artery, M2

## Abstract

**Background:**

Although the benefits of aspiration thrombectomy for treating acute ischemic stroke caused by proximal large vessel occlusion have been established, fewer data are available for evaluating aspiration thrombectomy of distal occlusion. The objective of this study was to evaluate, by means of prospectively collected data, the safety and efficacy of aspiration thrombectomy in patients with M2 middle cerebral artery (MCA) occlusion.

**Methods:**

This study is a subset analysis of a global prospective multicenter observational registry that included patients who presented with either anterior or posterior large vessel occlusion and were eligible for mechanical thrombectomy using the Penumbra System including the Penumbra 3D Revascularization Device. For this analysis, all patients in the registry with M2 MCA occlusion were included.

**Results:**

Of the 650 patients in the registry, 113 (17.4%) had M2 MCA occlusion. The rate of a modified treatment in cerebral infarction score of 2b to 3 after the procedure was 79.6% (90/113), the rate of a modified Rankin Scale score of 0–2 at 90 days was 72.5% (79/109), and the all-cause mortality rate at 90 days was 8.8% (10/113). Device-related serious adverse events occurred in one patient (0.9%) within 24 h and in two patients (1.8%) overall. Procedure-related serious adverse events occurred in four patients (3.5%) within 24 h and in six patients (5.3%) overall (nine events).

**Conclusion:**

For appropriately selected patients, aspiration thrombectomy for acute ischemic stroke due to M2 MCA occlusion was safe and effective, with high rates of technical success and good functional outcome.

## Introduction

Since publication of the pivotal trials establishing the overwhelming efficacy of thrombectomy over medical management for emergent large vessel occlusion (LVO) in acute ischemic stroke (AIS) (1), evaluation of patients outside of the original inclusion criteria and of advances in technique has been ongoing. Although the benefits of aspiration thrombectomy for treating AIS caused by proximal LVO have been established (2, 3), fewer data are available for evaluating aspiration thrombectomy for occlusion of more distal vessels (4). Current guidelines on treating AIS state that mechanical thrombectomy may be a reasonable treatment for distal middle cerebral artery (MCA) occlusion, but insufficient data exist to make this a strong recommendation (5, 6). Furthermore, no recommendations are available specifically on treating distal occlusion with aspiration thrombectomy (5–7).

The M2 segment of the MCA (M2 MCA) is the second most common LVO site (8). In the treatment of patients with M2 MCA occlusion, outcomes appear to be better for endovascular therapy (9), and for mechanical thrombectomy in particular (10, 11), than for best medical care. In studies comparing mechanical thrombectomy (11, 12) or aspiration thrombectomy (13–16) between MCA occlusion sites, fewer data are available for M2 MCA occlusion than for M1 MCA occlusion. The objective of this study was to evaluate, by means of prospectively collected data, the safety and efficacy of aspiration thrombectomy in patients with M2 MCA occlusion.

## Methods

This study is a subset analysis of a global prospective multicenter observational registry that included patients who presented with either anterior or posterior LVO and were eligible for mechanical thrombectomy using the Penumbra System including the Penumbra 3D Revascularization Device (Penumbra, Inc.) (17). This registry, COMPLETE (International Acute Ischemic Stroke Registry with the Penumbra System Aspiration Including the 3D Revascularization Device), was registered with ClinicalTrials.gov (NCT03464565) and enrolled patients from July 2, 2018, to October 9, 2019. For this analysis, all patients in the registry with M2 MCA occlusion were included. The M2 MCA was defined as the continuation of the distal M1 trunk beyond a holotemporal or posterior temporal branch (18).

Inclusion criteria for the registry were that the patient be at least 18 years of age, have a pre-stroke modified Rankin Scale score (mRS) of 0 or 1, experience AIS secondary to intracranial LVO and eligible for mechanical thrombectomy using the Penumbra System, have planned frontline treatment with the Penumbra System, and have a signed informed consent form per Institutional Review Board/Ethics Committee policy. Exclusion criteria were the patient's having any comorbid disease or condition expected to compromise survival or ability to complete follow-up assessments through 90 days or the patient's currently participating in an investigational clinical trial that would confound registry endpoints (patients in observational, natural history, or epidemiological studies not involving intervention were eligible for inclusion).

Baseline patient data collected were age, sex, race (collected only for patients in the United States), medical history, pre-stroke mRS, Alberta Stroke Program Early CT Score (ASPECTS), National Institutes of Health Stroke Scale score (NIHSS), side of occlusion, whether the patient was transferred from another hospital, whether intravenous tissue plasminogen activator (tPA) was given before the procedure, stroke onset, time from AIS onset to hospital admission, time from AIS onset to arterial puncture, and time from admission to arterial puncture.

The Penumbra System was used as the first line of treatment. The Penumbra System provides aspiration thrombectomy either alone (A Direct Aspiration First Pass Technique [ADAPT]) or in combination with the 3D Revascularization Device (ADAPT + 3D) (17). Commercial stent retrievers were allowed as a rescue treatment. The devices used for the frontline procedure and for additional attempts were recorded, as were the number of additional interventions and the times from onset and arterial puncture to a modified treatment in cerebral infarction score (mTICI) of 2b to 3 (mTICI 2b-3) or to the final angiogram.

The primary efficacy outcomes were the rates of revascularization of the target vessel (defined as mTICI 2b-3) after the procedure and an mRS of 0–2 (mRS 0–2) at 90 days. The primary safety outcome was the all-cause mortality rate at 90 days. The secondary efficacy outcomes were the rates of mTICI of 2c to 3 (mTICI 2c-3) and mTICI of 3 (mTICI 3) after the procedure. The secondary safety outcomes were the rates of device-related and procedure-related serious adverse events (SAEs) within 24 h and overall, embolization in new or uninvolved territory as seen on the final angiogram at the end of the procedure, intracranial hemorrhage (ICH) within 24 h, symptomatic ICH within 24 h of the procedure, vessel perforation, and vessel dissection.

Outcomes were adjudicated by an imaging core lab and two independent medical reviewers. The imaging core lab reviewed angiograms to determine treatment location, mTICI after each pass, and embolization in new or uninvolved territory at the end of the procedure. The imaging core lab also reviewed the pre-procedure CT scan to determine ASPECTS and reviewed the 24-h CT scan for symptomatic ICH by using European Cooperative Acute Stroke Study (ECASS) criteria. The independent medical reviewers adjudicated safety events on the basis of established criteria for safety endpoints, reviewed and adjudicated clinical events related to the device, and reviewed and ruled on any deaths.

Data were presented as descriptive statistics, including number of observations, mean and standard deviation or median and IQR for continuous variables, and percentages and counts for categorical variables. Analyses were performed by using SAS (version 9.4, SAS Institute).

## Results

### Baseline characteristics

Of the 650 patients in the registry, 113 (17.4%) had M2 MCA occlusion (Table 1). Average patient age was 71.1 years (SD 14.0), and 53.1% of patients were female. Most patients in the registry were from the United States, with the most prevalent race of those patients being White (66/82, 80.5%). The most common medical history characteristics were hypertension (74.3%), cardiovascular/vascular disease (61.9%), hyperlipidemia (45.1%), and atrial fibrillation (40.7%). A pre-stroke mRS of 1 was present in 30 patients (26.5%). The median pre-procedure ASPECTS was 9 (IQR 8–10) and the median pre-procedure NIHSS was 10 (IQR 6–16). The occlusion was on the left side in 74 patients (65.5%). Approximately half (49.6%) of the patients were transferred from another hospital. Intravenous tPA was administered before the procedure in 65 patients (57.5%). The stroke onset was witnessed in 66 patients (58.4%), unwitnessed in 32 patients (28.3%), and present upon waking up in 15 patients (13.3%). The median time from AIS onset to hospital admission was 3.0 h (IQR 1.2–5.7), the median time from AIS onset to arterial puncture was 4.8 h (IQR 3.3–8.1), and the median time from hospital admission to arterial puncture was 79 min (IQR 42–122).

**Table 1:** Baseline characteristics of patients treated with aspiration thrombectomy for acute ischemic stroke due to M2 middle cerebral artery occlusion.

*N*	113
Age, years	71.1 ± 14.0
Female	53.1% (60/113)
**Race (collected only for patients in the United States)**	
Asian	3.7% (3/82)
Black or African American	12.2% (10/82)
White	80.5% (66/82)
Not reported	3.7% (3/82)
**Medical history**	
Atherosclerosis	14.2% (16/113)
Intracranial	7.1% (8/113)
Extracranial—carotid	11.5% (13/113)
Extracranial—vertebral	2.7% (3/113)
Cardiovascular/vascular disease	61.9% (70/113)
Atrial fibrillation	40.7% (46/113)
Diabetes	24.8% (28/113)
Headaches/migraines	5.3% (6/113)
Hyperlipidemia	45.1% (51/113)
Hypertension	74.3% (84/113)
Renal failure	4.4% (5/113)
Seizures	0.9% (1/113)
**Presentation**	
Pre-stroke modified Rankin Scale score	
0	73.5% (83/113)
1	26.5% (30/113)
Alberta Stroke Program Early CT Score^a^	9 [8–10] (*n* = 112)
National Institutes of Health Stroke Scale score	10 [6–16]
Left-sided occlusion^a^	65.5% (74/113)
Transferred from another hospital	49.6% (56/113)
Intravenous tissue plasminogen activator given before procedure	57.5% (65/113)
Stroke onset	
Witnessed	58.4% (66/113)
Unwitnessed	28.3% (32/113)
Present upon waking up	13.3% (15/113)
**Process times**	
Time from onset to hospital admission, h	3.0 [1.2–5.7] (*n* = 108)
Time from onset to arterial puncture, h	4.8 [3.3–8.1] (*n* = 108)
Time from admission to arterial puncture, min	79 [42–122] (*n* = 106)

Continuous variables are reported as mean ± SD or as median [IQR] and categorical variables are reported as percentage (n/N).

a Core lab–adjudicated else treating physician–adjudicated.

### Procedural information

Access was femoral in almost all patients (97.3%; Table 2). The frontline procedural technique was aspiration alone in 68 patients (60.2%) and was aspiration plus the 3D Revascularization Device in 43 patients (38.1%). When aspiration alone was the frontline procedure, the most frequently used aspiration catheters were ACE68 (*n* = 19), JET7 (*n* = 16), and JETD and 3MAX (*n* = 13 each). When aspiration plus the 3D Revascularization Device was the frontline procedure, the most frequently used aspiration catheters were JET7 (*n* = 15), JETD (*n* = 11), and ACE68 (*n* = 11). In 56 patients (49.6%), only one pass was required to achieve mTICI 2b-3. The most frequently performed interventions after the first pass were aspiration alone (*n* = 50), 3D Revascularization Device (*n* = 24), and aspiration plus the 3D Revascularization Device (*n* = 21). Median time from onset to mTICI 2b-3 or to the final angiogram was 5.5 h (IQR 3.8–8.5), and median time from arterial puncture to mTICI 2b-3 or to the final angiogram was 31 min (IQR 19–45.5).

**Table 2:** Procedural information for patients treated with aspiration thrombectomy for acute ischemic stroke due to M2 middle cerebral artery occlusion.

**Type of arterial puncture**	
Femoral access	97.3% (110/113)
Other carotid access	2.7% (3/113)
**Frontline procedural technique**	
Aspiration alone	60.2% (68/113)
JET7	23.5% (16/68)
JETD	19.1% (13/68)
ACE68	27.9% (19/68)
ACE64	1.5% (1/68)
ACE60	5.9% (4/68)
4MAX	2.9% (2/68)
3MAX	19.1% (13/68)
Aspiration plus 3D	38.1% (43/113)
JET7 + 3D	34.9% (15/43)
JETD + 3D	25.6% (11/43)
ACE68 + 3D	25.6% (11/43)
ACE64 + 3D	2.3% (1/43)
ACE60 + 3D	7.0% (3/43)
4MAX + 3D	2.3% (1/43)
3MAX + 3D	2.3% (1/43)
Other	1.8% (2/113)
**Number of passes**	
1	49.6% (56/113)
2	22.1% (25/113)
3	14.2% (16/113)
4 or more	14.2% (16/113)
**Interventions performed after the first pass on target vessel or remaining clot** ^a^	
Aspiration alone	44.2% (50/113)
3D	21.2% (24/113)
Aspiration plus 3D	18.6% (21/113)
Stent retriever from another manufacturer	10.6% (12/113)
Other	8.8% (10/113)
Aspiration plus stent retriever from another manufacturer	6.2% (7/113)
**Procedure times**	
Time from onset to mTICI 2b-3 or final angiogram, h^b^	5.5 [3.8–8.5] (*n* = 107)
Time from arterial puncture to mTICI 2b-3 or final angiogram, min^b^	31 [19–45.5] (*n* = 112)

Continuous variables are reported as median [IQR] and categorical variables are reported as percentage (n/N).

3D, 3D Revascularization Device; mTICI, modified treatment in cerebral infarction score.

a Patients may have more than one type of additional intervention.

b Core lab–adjudicated.

### Outcomes

The rate of mTICI 2b-3 after the procedure was 79.6% (90/113), the rate of mRS 0–2 at 90 days was 72.5% (79/109, four patients lost to follow-up), and the all-cause mortality rate at 90 days was 8.8% (10/113; Table 3). The rate of mTICI 2c-3 after the procedure was 64.6% (73/113) and the rate of mTICI 3 after the procedure was 46.9% (53/113).

**Table 3:** Outcomes for patients treated with aspiration thrombectomy for acute ischemic stroke due to M2 middle cerebral artery occlusion.

**Primary outcomes**	
mTICI 2b-3 after procedure^a^	79.6% (90/113)
mRS 0–2 at 90 days	72.5% (79/109)
All-cause mortality at 90 days	8.8% (10/113)
**Secondary outcomes, by patient**	
mTICI 2c-3 after procedure	64.6% (73/113)
mTICI 3 after procedure^a^	46.9% (53/113)
Device-related SAEs within 24 h	0.9% (1/113)
Device-related SAEs, all	1.8% (2/113)
Procedure-related SAEs within 24 h	3.5% (4/113)
Procedure-related SAEs	5.3% (6/113)^b^
Embolization in new or uninvolved territory at end of procedure^a^	3.5% (4/113)
Intracranial hemorrhage within 24 h^a,c^	31.0% (35/113)
IPH	21.2% (24/113)
HI-1	8.0% (9/113)
HI-2	8.0% (9/113)
PH-1	4.4% (5/113)
PH-2	1.8% (2/113)
IVH	0.9% (1/113)
RIH	0.9% (1/113)
SAH	13.3% (15/113)
SDH	0.0% (0/113)
EDH	0.0% (0/113)
Symptomatic intracranial hemorrhage within 24 h	3.5% (4/113)
Vessel perforation	0.9% (1/113)
Vessel dissection	0.0% (0/113)

Variables are reported as percentage (n/N).

mRS, modified Rankin Scale score; mTICI, modified treatment in cerebral infarction score; SAE, serious adverse event.

a Core lab–adjudicated.

b Nine events.

c Multiple responses are allowed for the ECASS classifications.

Device-related SAEs occurred in one patient (0.9%) within 24 h and in two patients (1.8%) overall, with two events overall (Table 3). Procedure-related SAEs occurred in four patients (3.5%) within 24 h and in six patients (5.3%) overall, with nine events overall. In the seven patients in whom a device-related or procedure-related SAE occurred (one SAE was both device related and procedure related), three patients had hemorrhagic transformation (one with a subarachnoid hemorrhage [SAH] component) and one patient had SAH and ICH. Two of the patients who had hemorrhagic transformation had M2 and A2 occlusion; one of those patients also had a petrous segment dissection and an SAH component. The SAE in the patient who had SAH and ICH was considered both device related and procedure related and may have been caused by perforation of the 3D device during motion associated with the patient's ongoing vomiting, which caused the thrombectomy procedure to be stopped prematurely.

Embolization in new or uninvolved territory at the end of the procedure occurred in four patients (3.5%; Table 3). Intracranial hemorrhage within 24 h occurred in 35 patients (31.0%), and symptomatic ICH within 24 h occurred in four patients (3.5%). Vessel perforation occurred in one patient (0.9%) and vessel dissection occurred in no patients.

## Discussion

Although previous studies have indicated that outcomes of treating M2 MCA occlusion are better for mechanical thrombectomy than for best medical care (10, 11), guidelines on treating AIS do not yet strongly recommend mechanical thrombectomy as a treatment for distal MCA occlusion (5, 6). In this study, the authors used data from the COMPLETE registry to demonstrate that aspiration thrombectomy is a safe and effective technique for treatment of patients with AIS due to M2 MCA occlusion. These prospectively collected, independently adjudicated data from multiple international centers support the use of mechanical thrombectomy for acute M2 MCA occlusion. Importantly, almost 50% of the patients required only one pass of aspiration alone or aspiration plus 3D as the frontline procedural technique, and more than 70% of patients had a good functional outcome (mRS 0–2) at 90 days. Our results are corroborated by results from previous studies that reported the results of aspiration thrombectomy for M2 MCA occlusion and used prospectively collected data (Supplementary Table 1) (13, 14, 16, 19–24).

The outcomes from the current study were comparable to or better than the outcomes from three recent meta-analyses on mechanical thrombectomy of M2 MCA occlusion (Table 4) (12, 25, 26). These meta-analyses differed in their literature search time ranges; Findakly et al. (12) searched up to May 20, 2018, Li et al. (26) searched from after January 1, 2015, to March 2019 to include only second-generation thrombectomy devices, and Alexander et al. (25) searched from January 2015 to September 2019 to include only more recent technology. The mRS 0–2 rate at 90 days was considerably higher in the current study (72.5%) than in the meta-analyses [48.6% (25), 58.3% (12), and 59.3% (26)]. The revascularization rate after the procedure in the current study (79.6%, mTICI 2b-3) was comparable to those reported in the meta-analyses [72.8% (25), 75.4% (12), and 84.2% (26), TICI 2b-3 or mTICI 2b-3]. Likewise, the mortality rate at 90 days in the current study (8.8%) was also comparable to those reported in the meta-analyses [7.7% (26), 12.2% (12), and 16.3% (25)]. The rate of symptomatic ICH within 24 h was lower in the current study (3.5%) than in the meta-analyses [4.9% (25, 26) and 5.1% (12)].

**Table 4:** Recent meta-analyses on the results of mechanical thrombectomy for acute ischemic stroke due to M2 middle cerebral artery occlusion.

**Citation**	**Treatment or variable**	**No. patients**	**TICI or mTICI 2b-3 after procedure**	**mRS 0–2 at 90 days**	**Mortality at 90 days**	**sICH**
**Mechanical thrombectomy**						
Alexander et al. (25)	Mechanical thrombectomy	758 (11 articles)	72.8%	48.6%	16.3%	4.9%
Findakly et al. (12)	Mechanical thrombectomy	1,105 (15 articles)	75.4%	58.3%	12.2%	5.1%
Li et al. (26)	Mechanical thrombectomy	805 (seven articles)	84.2%	59.3%	7.7%	4.9%
**Mechanical thrombectomy vs. best medical care**						
Menon et al. (10)	Endovascular thrombectomy	67 (7 RCTs)	—	58.2%	11.9%	0.0%
	Best medical care	64 (7 RCTs)	—	39.7%	9.5%	7.9%
	Odds ratio	—	—	2.13^a^	1.29^a^	0^a^
				2.39^b^	1.33^b^	
	*P*-value	—	—	0.04^a^	0.66^a^	0.03^a^
				0.03^b^	0.66^b^	
Wang et al. (11)	Mechanical thrombectomy	531 (four articles)	—	60.8%^c^	5.7%^c^	4.9%^c^
	Best medical treatment	485 (four articles)	—	43.7%^c^	16.7%^c^	2.9%^c^
	Risk ratio	—	—	1.43	0.46	1.65
	*P*-value	—	—	0.011	0.022	0.286

mRS, modified Rankin Scale score; mTICI, modified treatment in cerebral infarction score; RCT, randomized controlled trial; sICH, symptomatic intracranial hemorrhage; TICI, treatment in cerebral infarction score.

a Unadjusted.

b Adjusted.

c Not reported by the authors; calculated here by using the double arcsine transformation and random effects model.

Two recent meta-analyses have concluded that for the treatment of patients with M2 MCA occlusion, outcomes appeared to be better for mechanical thrombectomy than for best medical care (Table 4) (10, 11). Menon et al. used data from the seven RCTs of the HERMES collaboration to compare patients with M2 MCA occlusion who were treated with endovascular thrombectomy vs. with best medical care (10). The rate of mRS 0–2 at 90 days was higher for endovascular treatment (58.2%) than for best medical care [39.7%; adjusted odds ratio (AOR) 2.39, adjusted *P* = 0.03], the mortality rate at 90 days was equivalent for endovascular treatment (11.9%) and best medical care (9.5%; AOR 1.33, adjusted *P* = 0.66), and the symptomatic ICH rate was lower for endovascular treatment (0.0%) than for best medical care (7.9%; unadjusted *P* = 0.03). The authors concluded that these results supported the efficacy of endovascular treatment over best medical care for patients with M2 MCA occlusion. Wang et al. used data from four reports to compare patients with M2 MCA occlusion who were treated with mechanical thrombectomy vs. with best medical treatment (11). The rate of mRS 0–2 at 90 days was higher for mechanical thrombectomy (60.8%) than for best medical treatment (43.7%; risk ratio [RR] 1.43, *P* = 0.011) and the mortality rate at 90 days was lower for mechanical thrombectomy (5.7%) than for best medical treatment (16.7%; RR 0.46, *P* = 0.022). For symptomatic ICH rate, no significant difference was detected between mechanical thrombectomy (4.9%) and best medical treatment (2.9%; RR 1.65, *P* = 0.286). The authors concluded that for patients with M2 MCA occlusion, mechanical thrombectomy was better than best medical treatment and was effective and safe.

Studies that have compared aspiration thrombectomy vs. stent retriever thrombectomy for M2 MCA occlusion generally found little difference in outcomes between the two techniques (Supplementary Table 2) (20–23, 27). In a *post-hoc* analysis of the ASTER trial, in which ADAPT and stent retriever thrombectomy for M2 MCA occlusion were compared, Gory et al. observed no significant difference between the groups for rates of additional intervention, revascularization after the procedure, mRS 0–2 at 90 days, or mortality at 90 days (20). Likewise, in a single-center study comparing manual aspiration thrombectomy and stent retriever thrombectomy for M2 MCA occlusion, Kim et al. observed no significant difference between the groups for rates of additional intervention, revascularization after the procedure, mRS 0–2 at 90 days, mortality at 90 days, or symptomatic ICH (21). In a multicenter study in which ADAPT and stent retriever thrombectomy for M2 MCA occlusion were compared, Mokin et al. reported rates of revascularization after the procedure, mRS 0–2 at 90 days, and mortality at 90 days that were similar between the groups (27). In an analysis of patients from the EITS Registry who had isolated M2 MCA occlusion, Muszynski et al. reported that no significant difference was detected between aspiration thrombectomy and stent retriever thrombectomy alone for rate of mRS 0–2 at 90 days or for symptomatic ICH rate and that the mortality rate at 90 days was lower for contact aspiration thrombectomy than for stent retriever thrombectomy (AOR 0.38, *P* = 0.042) (22). In a multicenter study in which aspiration thrombectomy, stent retriever thrombectomy, and a combined technique for M2 MCA occlusion were compared, Renieri et al. reported that no significant difference was detected between the groups for rate of mRS 0–2 at 90 days (23).

### Limitations and strengths

This study has several limitations. First, there is a lack of a randomized comparison medical care group in the COMPLETE registry. Secondly, functional outcomes might have been biased because patients could be enrolled after thrombectomy and mRS at 90 days was not assessed by an independent certified assessor. Additionally, not all consecutive cases were enrolled in the registry. Finally, all patients were treated with devices from a single manufacturer, which achieves homogeneity but might limit the external validity of our results. Strengths of this study include its prospective nature; the large sample size; and that the imaging outcomes were independently adjudicated by an imaging core lab and safety endpoints were adjudicated by independent medical reviewers, thus minimizing bias. Also, the multicenter and multi-operator nature of the COMPLETE registry decreased proficiency bias.

## Conclusion

Our results support that, for appropriately selected patients, aspiration thrombectomy for acute ischemic stroke due to M2 MCA occlusion might be a safe and effective option, with high rates of technical success and good 90-day functional outcome, accompanied by a favorable safety profile. The results from this study corroborate the results from previous studies in defining the results expected after mechanical thrombectomy for acute ischemic stroke due to M2 MCA occlusion.

## Data availability statement

The raw data supporting the conclusions of this article will be made available by the authors, without undue reservation.

## Ethics statement

The studies involving human participants were reviewed and approved by SSMSTL (SSM Health Care St Louis) IRB 1015 Corporate Square Dr. #150 St Louis, MO 63132 USA. Written informed consent for participation was not required for this study in accordance with the national legislation and the institutional requirements.

## Author contributions

All authors listed have made a substantial, direct, and intellectual contribution to the work and approved it for publication.
