# Supplementary material for: Aspiration thrombectomy of M2 middle cerebral artery occlusion to treat acute ischemic stroke: A core lab–adjudicated subset analysis from the COMPLETE registry and literature review
Source: Front Neurol. 2023 Mar 30;14:1076754. doi: 10.3389/fneur.2023.1076754 (PMC10100586; doi:10.3389/fneur.2023.1076754)
Supplement: Supplementary file 1 [file Table_1.pdf]

Supplementary Table 1 Literature review of individual studies, with prospectively collected data, on the results of aspiration thrombectomy for acute ischemic stroke due to M2 middle cerebral artery occlusion.

Citation	No. patients	Aspiration type	Frontline treatment included stent retriever	Additional inter-ventions (rescue)	mTICI 2b-3 after procedure	mTICI 2c-3 after procedure	mTICI 3 after procedure	mRS 0-2 at 90 days	Mortality at 90 days	ENT at end of procedure	sICH
Current study	113	Pump	38.1%	50.4%	79.6%	64.6%	46.9%	72.5% (79/109)	8.8%	3.5%	3.5%
	68		0%	48.5%	85.3%	67.6%	51.5%	83.1% (54/65)	4.4%	1.5%	2.9%
	43		100%	53.5%	69.8%	59.5%	40.5%	57.1% (24/42)	16.3%	4.7%	4.7%
Alawieh et al 2019 (13)	122[a]	Unspecified	0%	32.8%	---	63.9%[b]	---	38.7%	14.9%[c]	---	4.1%
Almallouhi et al 2019 (19)	6	Pump	0%	16.7%	83.3%	16.7%	---	---	---	---	0.0%
Brehm et al 2019 (14)	10	Unspecified	0%	Sometimes[d]	70.0%	20.0%	10.0%	---	---	0.0%	---
Brehm et al 2019 (14)	12	Pump and manual[e]	100%[e]	Rarely[f]	91.7%	33.3%	25.0%	---	---	8.3%	---
Gory et al 2018 (20)	48	Pump	0%	31.3%	89.6%	54.2%	35.4%	54.4% (25/46)	19.6% (9/46)	4.2%	6.3%
Kim et al 2017 (21)	25	Manual	0%	20.0%	72.0%[b]	---	---	84.0%	0.0%	0.0%	4.0%
Muszynski et al 2022 (22)	458	Unspecified	CA alone: 46.7%<br>Combined: 42.5%<br>SR alone: 10.7%	---	---	---	---	51.9%	19.6%	2.0%	9.3%
Navia et al 2020 (16)	37	Pump	5.4%	21.6%	91.9%	---	---	70.3%	2.7%	---	2.7%
Renieri et al 2022 (23)	93	Unspecified	0%	---	---	---	---	53.3% (49/92)	---	---	---
	239	Unspecified	100%	---	---	---	---	65.2% (137/210)	---	---	---
Yoshimoto et al 2021 (24)	15	Pump[g]	100%[g]	---	---	---	---	46.7%	6.7%	0.0%	0.0%

CA, contact aspiration; ENT, embolization in new or uninvolved territory; mRS, modified Rankin Scale score; mTICI, modified treatment in cerebral infarction score; sICH, symptomatic intracranial hemorrhage; SR, stent retriever; TICI, treatment in cerebral infarction score.
[a]Results from 65 patients with M2 superior segment occlusion and 57 patients with M2 inferior segment occlusion were averaged.
[b]TICI.
[c]Estimated from figure.
[d]Not reported; 30.6% of all occlusion sites in larger total dataset.
[e]Stent-retriever assisted vacuum-locked extraction (SAVE) technique.
[f]Not reported; 0.02% of all occlusion sites in larger total dataset.
[g]Blind exchange with mini-pinning (BEMP) technique.

Supplementary Table 2 Literature review of individual studies comparing first pass aspiration thrombectomy vs first pass stent retriever thrombectomy for acute ischemic stroke due to M2 middle cerebral artery occlusion.

**Citation**	**First pass thrombectomy type or statistic**	**No. patients**	**Additional inter-ventions (rescue)**	**TICI or mTICI 2b-3 after procedure**	**mTICI 2c-3 after procedure**	**TICI 3 or mTICI 3 after procedure**	**mRS 0-2 at 90 days**	**Mortality at 90 days**	**ENT at end of procedure**	**sICH**
Gory et al 2018 (20)	ADAPT	48	31.3%	89.6%	54.2%	35.4%	54.4% (25/46)	19.6% (9/46)	4.2%	6.3%
	Stent retriever	31	19.4%	83.9%	54.8%	41.9%	50.0% (15/30)	3.3% (1/30)	0.0%	3.2%
	Risk ratio	---	1.17	1.60	1.03	0.86	1.07	---	---	---
	P-value	---	0.38	0.36	0.90	0.36	0.84	0.078	---	---
Kim et al 2017 (21)	Manual aspiration	25	20.0%	72.0%	---	---	84.0%	0.0%	0.0%	4.0%
	Stent retriever	16	12.5%	87.5%	---	---	75.0%	6.2%	0.0%	0.0%
	P-value	---	0.685	0.441	---	---	0.689	0.390	---	0.999
Mokin et al 2017 (27)	ADAPT	51	29.4%	84.3%	---	58.8%	52.9%	15.7%	---	---
	Stent retriever	62	6.5%	87.1%	---	51.6%	59.7%	21.0%	---	---
Muszynski et al 2022 (22)	Unspecified aspiration	213	---	---	---	---	---	---	---	---
	Stent retriever	49	---	---	---	---	---	---	---	---
	Adjusted odds ratio	---	---	---	---	---	1.95	0.38	---	0.66
	P-value	---	---	---	---	---	0.119	0.042	---	0.444
Renieri et al 2022 (23)	Unspecified aspiration	93	---	---	---	---	53.3% (49/92)	---	---	---
	Stent retriever	133	---	---	---	---	62.2% (74/119)	---	---	---
	Combined	239	---	---	---	---	65.2% (137/210)	---	---	---
	P-value	---	---	---	---	---	0.142	---	---	---

ADAPT, a direct aspiration first pass technique; ENT, embolization in new or uninvolved territory; mRS, modified Rankin Scale score; mTICI, modified treatment in cerebral infarction score; sICH, symptomatic intracranial hemorrhage; TICI, treatment in cerebral infarction score.
